# Acknowledging the Role of Community Health Workers in Providing Essential Healthcare Services in Rural India-A Review

**DOI:** 10.7759/cureus.29372

**Published:** 2022-09-20

**Authors:** Poonam S Kalne, Pooja S Kalne, Ashok M Mehendale

**Affiliations:** 1 School of Epidemiology and Public Health, Datta Meghe Institute of Medical Sciences, Wardha, IND; 2 Department of Toxicology, Chaitanya Ayurved College and Hospital, Bhusaval, IND; 3 Department of Community Medicine, Jawaharlal Nehru Medical College, Datta Meghe Institute of Medical Sciences, Wardha, IND

**Keywords:** adolescent health, maternal and child health, rural health, primary health care, community health workers

## Abstract

In underserved communities, the community health worker (CHW) concept has been employed to improve health and lessen unfavourable health consequences. In India's rural healthcare delivery system, auxiliary nurse midwives (ANMs), accredited social health activists (ASHA workers), and Anganwadi workers (AWWs) are the primary field-level frontline officials who come into direct contact with the population. They bear a large portion of the burden of carrying out health services. This review investigated the various contributions made by these CHWs, ANMs, ASHA workers, and AWWs to the advancement of basic healthcare in Indian rural areas. The goal of reviewing this paper was to learn more about what CHWs do to provide the target demographic with high-quality healthcare. A thorough literature search was conducted using crucial databases including PubMed, Google, and Google Scholar. Recent studies were examined to determine how well CHWs perform essential healthcare services in low and middle-income nations. Numerous studies demonstrate how their work has a good effect on society. The length of time CHWs spend at work each day and how well they perform as a whole depends on several variables. This review study showed that, globally, there is a growing interest in CHWs' performance. In terms of incentives, pay, and training expenses, CHWs are thought to be a more affordable option than other types of health workers. They are recognised as the main factors in providing promotive, preventive, curative and rehabilitative healthcare services, achieving enhanced neonatal and maternal health and the development of children and adolescents. The current review also examined previous studies on the work done by CHWs and their potential benefits for enhancing primary healthcare in rural India. It focused on the routine work done by these health workers to increase service accessibility and access to high-quality healthcare, particularly for individuals living in rural areas. Hence, it is necessary to evaluate the functions and general status of community health workers (CHWs), as well as recognise their role, to improve their efficiency in providing basic healthcare services to society and make necessary changes in the future.

## Introduction and background

The Indian government established the Rural Health Scheme in 1977 in response to the Shrivastav Committee's recommendations, with the intention of "putting people's health in people's hands" (1975). The Sub-center, Primary Health Centre (PHC), and Community Health Centre (CHC) make up the three-tiered structure of primary healthcare infrastructure in rural areas of India. At the Alma Ata International Health Conference in 1978, the World Health Organization (WHO) set the worldwide, societal objective of "Health for All by 2000 AD" [1,2]. Accredited social health activists (ASHAs), auxiliary nurse midwives (ANMs), and Anganwadi workers (AWWs) are considered the main frontline health workers in India. The fundamental tenets of primary healthcare are equitable resource distribution and universal coverage. Primary healthcare is delivered in rural areas not by medical staff but by non-medical staff known as community health workers (CHWs). They raise awareness and eventually strengthen the basic healthcare system. Their main responsibility is to enhance mother and child health. In many different countries, CHWs have very distinct responsibilities as per their training programs. Many nations struggle to maintain and develop the literate health professionals required to provide the community with good health. As a result, particularly in low-income countries, there is sometimes a lack of trained community health practitioners to shoulder a whole load of lowering morbidity and mortality in society [3]. In addition to providing very basic health services, community health workers play a far more significant role [4]. Their most significant promotional and developmental role is to form a bridge between health services and the community. There is increasing interest in the performance of CHWs globally [5]. They are considered to be a less expensive alternative as compared to other groups of health workers, mainly in terms of incentives and salaries, as well as training costs. They have been acknowledged as the primary factors in achieving better neonatal, mother, child, and adolescent health and development [6]. The government's primary objective at the moment is to offer healthcare that is inexpensive, accessible, accountable, effective, equitable, and reliable, especially for the nation's impoverished and most vulnerable rural residents [1]. The many roles that these community health workers (ANMs, ASHA workers, and AWWs) play in delivering primary healthcare services to the target population in rural India were reviewed and appreciated. This review study explains why the Integrated Child Development Service (ICDS) scheme requires its local female health professionals, known as Anganwadi workers (AWWs), to have finished their education up to 10th grade. By acting as CHWs to meet the needs of women and children, the AWWs and the other senior-level staff members, ANMs, have considerably improved the Indian health care delivery system since 1975 [7]. To promote access to and utilisation of health services, the Government of India (GOI) introduced the National Rural Health Mission (NRHM) programme in 2005. To assist AWWs and ANMs in their work, one of their crucial components entailed recruiting local women to participate in training to become accredited social health activists (ASHA workers) under the Ministry of Women and Child Development. They are local women, typically between the ages of 25 and 45, and have had at least eight years of education [7,8].

According to available studies, CHWs are crucial to the healthcare system's ability to deliver high-quality community-based services required by rural communities. The lack of human resources, inadequate infrastructure, and poor care are blamed for India's high rates of newborn and maternal mortality. This might be because the country has a small health budget and it amounts to less than 2% of all government spending as a proportion of the Gross Domestic Product (GDP) [9]. Furthermore, the literature shows that, due to these financial limitations, it is essential to examine the work processes of CHWs to pinpoint the promoters of these processes and the difficulties they encounter to improve the country's healthcare system as a whole. The National Rural Health Mission (NRHM) of the Government of India was founded to support rural people that are disadvantaged and underserved. This group of functionaries may work together to alter the desired behaviour and reduce the social delays that contribute to maternal and paediatric mortality [3,10,11]. Additionally, it will promote a sense of ownership and participation in the community. The programme will take years for it to bring about noticeable improvements at the community level and become a reality [12].

This review will help know the actual roles and responsibilities of these community health workers. It will be useful to the overall community, government, policymakers, health planners, national programs, researchers, programme managers, and the public health system of India to know more about the actual role, functions, and contributions of these CHWs in improving the health status of the community. It will also help in knowing the promoters and challenges faced by them while providing basic health needs to the community, which will eventually become useful in making efforts to improve their work and status in society and thus build a healthy nation.

Methodology

In the current study, we used databases like PubMed and Google Scholar to conduct targeted literature searches to find pertinent original research and review papers. "Community health professionals", "primary healthcare", "rural health", "maternal and child health", and "adolescent health" were the main search phrases. Our research was based on available material that was located after a careful search across PubMed and Google Scholar. Reviewing the titles and abstracts from these searches, obtaining the full texts of pertinent manuscripts, and looking through reference lists to find additional manuscripts that should be reviewed were all done. Considering that this typical review is not a systematic review, no guidelines from Assessment of Multiple Systematic Reviews (AMSTAR) or Preferred Reporting Items for Systematic Reviews and Meta-Analyses (PRISMA) were used.

## Review

The National Rural Health Mission (NRHM) in India has as one of its key goals to increase the accessibility of workers in rural areas. A programme called "community health workers" exists to accomplish this. Community health workers (CHWs) are paid volunteers who work in neighbourhoods to promote the development of healthcare systems and services for areas with limited resources and challenging access [10]. The goal of this review paper was to acknowledge the diverse roles of CHWs in improving the health status of the community, raising awareness and understanding among the public, and improving primary health care services to meet their needs. The literature discusses their function in offering fundamental healthcare services to the underprivileged population in rural India. AWWs plan different nutrition-related initiatives and other helpful activities. As directed by an AWW, an ASHA worker performs health care duties, such as conducting workshops to increase understanding of child care, a healthy diet, prenatal care, personal hygiene, and the importance of immunization. An ANM provides services in the following areas: family planning; health, nutrition, and education; sanitation; environmental protection; immunisation for the prevention of infectious illnesses; care for minor injuries; and first aid in times of emergencies and disasters [1,2,13]. Knowing more about their jobs and work schedules is vital to taking the appropriate actions because they are the main frontline health workers promoting the development of a healthy society. This traditional review elaborates that, in the rural community, these CHWs offer preventive, promotive, curative, and rehabilitative health facilities. Additionally, it claims that the welfare of mother and child is a vital public health issue that reflects each nation's socioeconomic standing. ANMs, ASHA workers, and AWWs are among the frontline staff in the primary healthcare delivery system who are expected to work in this area to enhance reproductive and child health (RCH) indicators [14].

Maternal and child health care services provided by community health workers (CHWs)

ANMs, ASHA workers, and AWWs are frontline public health workers who are well-liked in their communities and who are educated about the area they serve. According to peer-reviewed studies, CHWs can influence several mother and child health outcomes, including newborn birth weight [15]. It is well established that socioeconomic factors, such as those relating to birth, growth, living, working, and ageing, as well as variations in access to health care, are the main causes of ethnic and racial disparities in mother and infant health [16,17].

The ASHA programme is essential for offering outreach services for Reproductive, Maternal, Neonatal, and Child Health and Nutrition (RMNCHN). ASHA workers and two additional female CHW cadres-auxiliary nurse midwives (ANMs) and Anganwadi workers (AWWs)-perform complementary duties in the field [18]. AWWs and ASHA workers work together to coordinate health days once or twice a month. They update the list of eligible couples and young children and mobilise nursing mothers and infants for nutrition support. They also bring beneficiaries from the village to the Anganwadi centre on designated days for immunization; facilitate institutional deliveries; provide basic medications; and perform health check-ups on health days [1]. Through the integrated child development service (ICDS) program, AWWs offer a range of services, including complementary nutrition to expectant and breastfeeding women and children aged 3-6 years; iron and folic acid supplements; immunization; health examinations; mild disease treatment; and referral services for mothers and children; women's non-formal health and nutrition education; children's preschool education; and the convergence of other supportive services like water, sanitation, etc [19]. ANMs are recognised as multipurpose workers (MPWs) with a wide range of duties, including aiding AWWs and training ASHA workers. They assist community health officers (CHOs) and medical officers (MOs) with outpatient department (OPD) operations and remain available to respond to emergencies [20-22]. Some receive additional training for the placement of intrauterine devices, while others receive it for managing delivery difficulties and referring women who have them to higher levels of care. ANMs inform ASHA workers when and where the outreach sessions will be taking place and assist them in maintaining a list of couples qualified for family planning. They also assist ASHA workers in organising health days at the Anganwadi centre by taking part in them [2].

To lower disease and mortality rates among women and children across the nation, the home-based newborn care (HBNC) approach directs CHWs to recognise and treat neonatal morbidities like hypoxia, preterm birth, fever, umbilical sepsis, low birth weight, and other conditions as soon as they arise [23]. To identify patients with vector-borne diseases like malaria, dengue, etc., ANMs and ASHA workers undertake container surveys. They also find patients with leprosy, tuberculosis (TB), coronavirus disease (COVID-19), and other communicable diseases. ANMs, ASHA workers, and AWWs regularly visit beneficiaries' homes to meet their requirements for basic health care, including those of expectant and lactating mothers, newborns, infants, and low-birth-weight babies. They maintain various records ranging from births, deaths, weights, heights, mid-upper arm circumferences, immunisation status, and beneficiaries under different Yojna’s (schemes) to make the information available whenever needed [6,16,24].

Adolescent healthcare services provided by community health workers (CHWs)

Adolescents account for 22.3% of the global population. By understanding the difficulties experienced by young women, the National Rural Health Mission (NRHM) offers them adolescent health services. The Reproductive, Maternal, Neonatal, Child, and Adolescent Health (RMNCH+A) Plan was a 2013 initiative that aimed to address the leading causes of death for women and children. The RMNCH+A strategy aims to increase awareness of the "continuum of care" throughout different life phases [25]. According to the WHO, adolescence is the decade between the ages of 10 and 19, and it is characterised by fast growth and development on the physical, psychological, and social fronts as a child reaches adulthood [26]. Establishing adolescent-friendly health clinics (AFHCs) and providing quality adolescent health care services in the community is the new initiative taken by the Indian government to provide basic health care services. Through these clinics, counselling of adolescents regarding their health issues is done [27]. An ANM, along with an ASHA worker and AWW, makes efforts to improve adolescents' nutrition status; distributes iron-folic acid tablets and deworming tablets; promotes menstrual hygiene schemes; raises awareness against sexually transmitted diseases (STDs), HIV/AIDS, and contraception; and conducts preventive health check-ups. AWWs conduct Kishori Sabhas to spread awareness among adolescent girls and Kishori Shakti Yojna (KSY) to empower them and enable them to take charge of their lives [25]. Education and counselling are given by CHWs to enhance knowledge, attitudes, and behaviours towards sexual and reproductive health, mental health, drug addiction, and non-communicable illnesses. Education in life skills is also provided in both formal educational settings and informal community settings [28].

Promotive, preventive, curative, and rehabilitative health care services provided by community health workers (CHWs)

The ANM cadre, which was established in the 1960s, is the group of village-level health workers with the most training and experience. The AWW programme has been a part of the healthcare system since the middle of the 1970s, and it is renowned in the fields of nutrition and child care. In 2005, the National Rural Health Mission (NRHM) unveiled the ASHA scheme, a brand-new cadre. ANM and AWW cadres, who are the newest and typically younger additions, supervise and support ASHA workers [2]. Table 1 represents the roles and responsibilities of all CHWs.

**Table 1:** Roles and responsibilities of CHWs ANM- Auxiliary nurse midwife ASHA- Accredited social health activist AWW- Anganwadi worker STDs- Sexually transmitted diseases TB- Tuberculosis ORS- Oral rehydration solution ANC- Antenatal care DOTs- Directly observed therapy, short course

Sr no.	Roles & responsibilities of ANMs [2, 28-33]	Roles & responsibilities of AWWs [19-34]	Roles & responsibilities of ASHA workers [2, 35]
1	Hold monthly or biweekly meetings with ASHA workers and serve as a reference person for ASHA training alongside the AWW.	Help the ASHA worker organise a health day every second or third week.	Raise community awareness and inform them about factors that affect health, such as diet, daily habits, lifestyle practices, and working situations.
2	Inform the ASHA workers when and where the outreach sessions will be taking place.	Help the ASHA workers carry out health-related education through health days.	Make efforts to make health care services available to society and to raise awareness about the importance of using them as soon as possible.
3	Assist ASHA workers in keeping a list of couples qualified for family planning, encouraging expectant mothers to attend antenatal care (ANC) sessions, and ensuring that they receive tetanus toxoid injections and iron supplements.	Enhancing the nutritional and health status of young children (0–6 years) and pregnant and lactating mothers.	Give women advice on how to get ready for pregnancy, have a safe delivery, care for their new-born, the importance of breastfeeding, complementary feeding and immunization, use contraception, and stay away from sexually transmitted diseases (STDs) and common illnesses.
4	Inform ASHA workers about oral contraceptive pill dosage instructions and side effects.	Make efforts to decrease new-born and child mortality rates and school dropout rates.	Encourage community involvement and access to medical care.
5	ASHA workers are trained by ANMs to recognise the early indications of pregnancy and labour so they can help expectant people seek out extra care as needed.	Establishing a solid basis for the child's optimal psychological, physical, and social growth.	Create a thorough local health plan with the community health and sanitation committee.
6	Let ASHA workers know when and where the initial and ongoing training will take place.	Improving maternal education and her ability to take care of her family's nutrition and health.	Make it convenient for children and expectant women to get medical care at a nearby hospital.
7	Assure that ASHA workers are paid for their work and for attending training.	Effective policy and executive coordination between various agencies and programs aiming to advance child development.	Give first aid for minor injuries and basic medical care for illnesses, including fevers and diarrhoea.
8	Assist ASHA workers in organising health days at the Anganwadi centre by taking part in them.	-	Give Directly observed therapy, short-course (DOTs) to patients with TB .
9	-	-	Bring supplies that will help the community in need (for example, condoms, iron and folic acid tablets, oral rehydration (ORS) packets, medicines for tuberculosis, chloroquine [in malaria-endemic areas], disposable delivery kits, and oral contraceptive pills).
10	-	-	Notify the medical system of any births, deaths, illness outbreaks, or unusual health issues.
11	-	-	As part of the Total Sanitation Campaign, promote the construction of toilets.
12	-	-	Care for newborns at home (a new role added in 2011).

Recommendations

Health initiatives targeting marginalised groups must include CHW training programs. These initiatives can successfully contribute when the right people are chosen, trained, and given continuing support. On the other hand, countless significant programmes have fallen short in the past as a result of exaggerated hopes and inadequate preparation.

Strategies to enhance the performance of community health workers (CHWs) 

Assuring community acceptance and membership, using appropriate practice-focused training techniques, and basing training on expected duties and responsibilities are important aspects of helping CHWs perform their roles and responsibilities. Managing and supervising CHWs is also important to improve their efficiency. Emphasizing career progression chances in CHW recruitment; increasing supervisors' reminders to CHWs about overdue assignments; offering good incentives for CHWs that are properly suited to their working hours, and effective CHW recruitment and selection for pre-service training may increase the performance of CHWs and the calibre of services offered. It is generally believed that by actively including the community being served in the recruitment of CHWs, the level of acceptance and trust for CHWs will increase.

## Conclusions

Our research indicates that the roles and responsibilities performed by CHWs, ANMs, ASHA workers, and AWWs are very crucial in providing basic healthcare services to the vulnerable population of India and should be acknowledged by the community and government. This will ensure that they will get respect, good status, and appreciation for their continued work in society. These health workers work at the lowest levels of the healthcare system with few incentives and without any holidays to improve the health status of the country with a sense of service to others. But their rights as employees are usually violated. The nurturing role that these CHWs play in the community is often neglected. Also, their work is considered inferior in the local community, where most of these CHWs are women. It is very common to overlook this gender issue in rural areas. This review will assist in recognising the important role played by these CHWs in building a healthy nation. It will provide detailed information about different jobs and activities performed by health workers, which will ultimately help in making necessary changes to make their work more efficient. To ensure CHWs' rights and status in society, it is necessary to reevaluate the fundamental qualities of CHWs and to clearly define their roles, responsibilities, and working hours. We also found that the government's health activities are facilitated when CHWs are involved in national programs.

Therefore, the modalities of governance need to be changed to encourage their integration as full participants in the public health team. Their work and diverse roles should be recognised in society so that they will be encouraged to perform their regular activities. Many arguments regarding motivation, efficacy, cost and quality assurance may be raised in opposition to any suggestion that CHWs be reclassified as workers. Some research papers show that the identification of a core set of skills, instruction, oversight, and infrastructure support will all be necessary. By acknowledging their role in this study, we suggest that the government and the general public may help CHWs to establish a safe, wholesome, happy, and constructive society in the future by honouring their contributions and supporting their ongoing work.
